# Ca^2+^ Release in Muscle Fibers Expressing R4892W and G4896V Type 1 Ryanodine Receptor Disease Mutants

**DOI:** 10.1371/journal.pone.0054042

**Published:** 2013-01-07

**Authors:** Romain Lefebvre, Claude Legrand, Linda Groom, Robert T. Dirksen, Vincent Jacquemond

**Affiliations:** 1 Centre de Génétique et de Physiologie Moléculaire et Cellulaire, CNRS UMR 5534 – Université Lyon 1, Villeurbanne, France; 2 Department of Pharmacology and Physiology, University of Rochester Medical Center, Rochester, New York, United States of America; University of Queensland, Australia

## Abstract

The large and rapidly increasing number of potentially pathological mutants in the type 1 ryanodine receptor (RyR1) prompts the need to characterize their effects on voltage-activated sarcoplasmic reticulum (SR) Ca^2+^ release in skeletal muscle. Here we evaluated the function of the R4892W and G4896V RyR1 mutants, both associated with central core disease (CCD) in humans, in myotubes and in adult muscle fibers. For both mutants expressed in RyR1-null (dyspedic) myotubes, voltage-gated Ca^2+^ release was absent following homotypic expression and only partially restored following heterotypic expression with wild-type (WT) RyR1. In muscle fibers from adult WT mice, both mutants were expressed in restricted regions of the fibers with a pattern consistent with triadic localization. Voltage-clamp-activated confocal Ca^2+^ signals showed that fiber regions endowed with G4896V-RyR1s exhibited an ∼30% reduction in the peak rate of SR Ca^2+^ release, with no significant change in SR Ca^2+^ content. Immunostaining revealed no associated change in the expression of either α1S subunit (Cav1.1) of the dihydropyridine receptor (DHPR) or type 1 sarco(endo)plasmic reticulum Ca^2+^ ATPase (SERCA1), indicating that the reduced Ca^2+^ release resulted from defective RyR1 function. Interestingly, in spite of robust localized junctional expression, the R4892W mutant did not affect SR Ca^2+^ release in adult muscle fibers, consistent with a low functional penetrance of this particular CCD-associated mutant.

## Introduction

Skeletal muscle cells contract in response to a rise in cytosolic Ca^2+^ due to the opening of the type 1 ryanodine receptor (RyR1) Ca^2+^ release channels in the SR membrane; RyR1 opening and closure occur through intimate conformational coupling processes with voltage-sensitive dihydropyridine receptor (DHPR)/Cav1.1 proteins in the transverse tubule (T-tubule) membrane. As a result, SR Ca^2+^ release is under tight control by the potential across the T-tubular membrane. The fact that several types of muscular disorders (e.g. malignant hyperthermia, central core disease, multi-minicore disease) result from either inherited or *de novo* mutations in the *RYR1* gene underscores the critical role that proper RyR1 Ca^2+^ release channel function plays in coordinating skeletal muscle performance (for review, see [1]–[3]). Among these disorders, central core disease (CCD) is associated with more than 100 *RYR1* mutations [4]. CCD is functionally characterized by hypotonia and muscle weakness. For still unknown reasons, the extent and severity of both clinical symptoms and pathological changes in muscle vary considerably between patients, even within the same family [5]–[7]. In addition, the actual incidence of the disease in the general population may be much higher than originally thought [7]. Thus, there is a critical need to provide a more comprehensive understanding of how specific mutations alter RyR1 function in skeletal muscle.

Expression studies in myotubes derived from RyR1-null (dyspedic) mice have revealed that mutations associated with a CCD phenotype that do not increase susceptibility for malignant hyperhermia (MHS) result in reduced SR Ca^2+^ release, independent of either enhanced SR Ca^2+^ leak or a reduction in SR Ca^2+^ content [8]–[10]. On the other hand, the same mutations promote SR Ca^2+^ leak and store depletion following heterologous expression [11] and in patient derived non-muscle cells [12]. Thus, it is essential that analysis of suspected CCD variants be conducted in muscle cells in which the release channel is under the intimate control of Cav1.1 channels in the T-tubule membrane. This is best achieved through the generation of knock-in mouse models for CCD, as was recently accomplished for the Y522S [13], [14] and I4898T CCD mutations in RyR1 [15]–[17]. Using muscle fibers from knock-in mice, Loy et al [18] demonstrated that the I4898T CCD mutation in RyR1 reduces SR Ca^2+^ release in skeletal muscle by producing a dominant-negative suppression of RyR1 Ca^2+^ ion permeation.

Although knock-in mouse models provide an incredibly powerful means for elucidating the primary defect of RyR1 disease mutations on skeletal muscle function, it is not possible to employ this approach for each of the more than 300 disease-associated RyR1 variants. We recently demonstrated that *in vivo* electroporation of cDNA constructs encoding green fluorescent protein (GFP)-tagged RyR channels provides an alternative approach to characterize the functional properties of the expressed channel in fully-differentiated adult skeletal muscle fibers [19], [20]. Specifically, using this approach we demonstrated consistent alterations in either voltage-sensitivity or in peak SR Ca^2+^ release in fibers expressing MHS or CCD associated RyR1 mutants, respectively [20]. Furthermore, a particularly powerful aspect of this approach is that exogenous channels are expressed locally within the muscle fibers such that direct comparisons can be made between the physiological activity of exogenously expressed channels with that of adjacent, native WT RyR1 channels present within the same cell [20].

In the present work, we used this approach to explore the functional properties of two CCD-associated RyR1mutants for which there is yet no data available in adult muscle fibers. These two CCD RyR1 mutants (R4892W and G4896V – numbered according to rabbit RyR1 sequence) affect residues lying either within (G4896V) or immediately adjacent (R4892W) to the RyR1 selectivity filter. The corresponding mutations in the human RyR1 sequence are R4893W and G4897V, respectively. In order to avoid confusion, numbering according to the rabbit RyR1 sequence (i.e. R4892W and G4896V) is used throughout rest of this study. We found that while both mutants markedly reduce voltage-gated Ca^2+^ release when co-expressed with WT RyR1 in dyspedic myotubes, voltage-gated Ca^2+^ release was significantly reduced only for the G4896V mutant following transient expression in muscle fibers of adult WT mice.

## Materials and Methods

### Ethics Statement

Experiments and procedures were conducted in accordance with the guidelines of the local animal ethics committee of the University of Rochester (2006–114R), University Lyon 1, of the French Ministry of Agriculture (87/848) and of the European Community (86/609/EEC); they were specifically approved by the animal ethics committes of University Lyon 1 and of University of Rochester.

### Preparation and expression of enhanced green fluorescent protein (EGFP)-tagged RyR1 mutants in dyspedic myotubes

The two identified RYR1 mutants (R4892W and G4896V) were introduced into both non-tagged and N-terminal EGFP-tagged versions of a full-length rabbit RYR1 complementary DNA (cDNA) (accession #X15750) using standard two-step site-directed mutagenesis. All sequences generated and modified by polymerase chain reaction were checked for integrity by sequence analysis. Myotubes were prepared from primary cultures of myoblasts obtained from skeletal muscle of newborn RYR1-null (dyspedic) mice as described previously [8], [21]. Expression of each non-tagged construct (WT, R4892W and G4896V) was achieved by direct microinjection of myotube nuclei with cDNA mixtures including CD8 (0.1 μg/μl) plus the appropriate RYR1 expression plasmid (0.5 μg/μl) [8], [21]. In co-expression experiments, nuclei of dyspedic myotubes were microinjected with a 1∶1 cDNA mixture (0.25 μg/μl each) of two plasmids (WT+R4892W or WT+G4896V). Expressing myotubes were identified 2–4 days after nuclear microinjection by incubation with CD8 antibody-coated beads as described previously [8], [21].

### Intracellular Ca^2+^ measurements in expressing myotubes

Intracellular Ca^2+^ measurements were obtained from Indo-1 AM-loaded myotubes as described previously [8], [21]. Briefly, myotubes grown on glass bottom dishes were loaded with 6 µM Indo-1AM for 1 hour at 37°C in a normal rodent Ringer's solution consisting of (in mM): 145 NaCl, 5 KCl, 2 CaCl_2_, 1 MgCl_2_, 10 HEPES, pH 7.4. Cytosolic dye within a small rectangular region of the expressing myotube was excited at 350±10 nm, fluorescence emission at 405±30 nm (F_405_) and 485±25 nm (F_485_) was measured at a 100 Hz sampling frequency with a photomultiplier detection system (Photon Technology International, Birmingham, NJ, USA), and results were presented as the ratio of 405 and 485 nm (F**_405_**/F**_485_**). A standard excitation-contraction (EC) uncoupling screening protocol was used to sequentially measure electrically-evoked Ca^2+^ release, agonist-induced RyR1 Ca^2+^ release and total SR Ca^2+^ content. This consisted of applying, via a local perfusion system, 30 seconds of control Ringer's solution followed by 10 electrically-evoked release events (1Hz), 10 seconds of Ringer's solution, 30 seconds of 500 µM 4-chloro-m-cresol (4-CMC), 30 seconds of Ringer's solution and 30 seconds of a Ca^2+^ store release cocktail consisting of 10 µM Ionomycin, 30 µM Cyclopiazonic acid (CPA) and 100 µM EGTA (ICE) in a Ca**^2+^** free Ringer solution (in mM): 145 NaCl, 5 KCl, 3 MgCl**_2_**, 10 HEPES, pH 7.4. Peak changes in intracellular Ca**^2+^** were expressed as ΔRatio (R**_agonist_** – R**_baseline_**). The peak value during 4-CMC application was always taken as the maximum value of the change in indo-1 ratio, whether this occurred during an oscillation or a slowly rising transient. The response to the ICE cocktail was previously shown to be a sensitive index of the SR Ca^2+^ store capacity with negligible contamination of Ca^2+^ from outside as the signal is strongly reduced by prior partial SR store depletion with a low concentration of CPA [15] and is further enhanced by subsequent exposure to 10 mM extracellular Ca^2+^ [22].

### Electroporation of EGFP-tagged RyR1 constructs in skeletal muscle of adult mice

Exogenous expression of RyR1 mutants by electroporation was performed in the *flexor digitorum brevis* (*fdb*) and interosseus muscles of 6–8 week-old Swiss OF1 male mice using a procedure described previously [19], [20], [23]. Specifically, mice were anaesthetized by isoflurane inhalation (3% in air, 300 ml.min^−1^) using a commercial delivery system (Univentor 400 Anaesthesia Unit, Uninventor, Zejtun, Malta). Twenty five microliters of a solution containing 2 mg/ml hyaluronidase dissolved in sterile saline was then injected into the footpads of each hind paw. Forty minutes later the mouse was re-anaesthetized by isoflurane inhalation. Plasmid DNAs (EGFP-G4896V or EGFP-R4892W) were then injected into the footpads of the animal at a concentration of 1.5 µg/µl in standard Tyrode solution. A 20 µl total volume of this solution was injected in different locations so as to target both the *fdb* and the interosseus muscles. Following the injection, two gold-plated stainless steel acupuncture needles connected to the electroporation apparatus were inserted under the skin, near the proximal and distal portion of the foot, respectively. The standard protocol used consisted in 20 pulses of 110 V/cm amplitude and 20 ms duration delivered at a frequency of 2 Hz by a BTX ECM 830 square wave pulse generator (Harvard Apparatus, Holliston, MA, USA). Experimental observations and measurements were carried out 2 weeks later.

### Preparation of isolated muscle fibers

Single fibers were isolated from *fdb* and interosseus muscles using a procedure described previously [24]. Briefly, mice were killed by cervical dislocation before removal of the muscles. Muscles were incubated in the presence of collagenase (Sigma, type 1) for 60 min at 37°C. Single fibers were then obtained by triturating the muscles within a 50 mm wide culture µ-dish (Ibidi, München, Germany). Fibers were partially insulated with silicone grease as described previously [24]. Briefly, fibers were embedded within silicone so that only a portion of the fiber extremity was left out of the silicone allowing whole-cell voltage clamp recordings to be restricted to the silicone-free extremity of the fiber. Expressing fibers were handled with silicone so that the fiber region exhibiting EGFP fluorescence was left out of the silicone. The fiber interior was then dialyzed with an intracellular solution containing 100 µM rhod-2, 20 mM EGTA and 8 mM CaCl_2_ (see Voltage clamp solutions). The internal solution was introduced within the voltage-clamp pipette, the tip of which was inserted through the silicone, within the insulated part of the fiber. In order to ease intracellular dialysis, the electrode tip was slightly crushed within the silicon-insulated portion of the fiber by moving it back and forth a few times in a non-gentle manner towards the bottom of the chamber. Intracellular equilibration of the solution was allowed for a period of 30 min with the holding voltage held at −80 mV, before initiating measurements. All experiments were performed at room temperature (20–22°C).

### Confocal fluorescence measurements on isolated fiber

Experiments were performed with a Zeiss LSM 5 Exciter confocal microscope (Carl Zeiss, Jena, Germany) equipped with a 63× oil immersion objective (numerical aperture 1.4). EGFP excitation was provided by the 488 nm line of an argon laser and a 505 nm long pass filter was used on the detection channel. For detection of rhod-2 fluorescence, excitation was from the 543 nm line of a HeNe laser and fluorescence was collected above 560 nm. One major aim of the experiments was to simultaneously record intracellular rhod-2 Ca^2+^ signals in both an EGFP-positive fiber region (i.e. enriched in exogenously expressed RyR1s) and adjacent EGFP-deprived regions where the direct contribution of exogenous RyR1s should be much less. To accomplish this objective, we worked specifically on imaging a silicone-free portion of the fiber that exhibited large and restricted EGFP signals. Intracellular Ca^2+^-related fluorescence changes were imaged by using the line-scan mode of the confocal system with the line set parallel to the longitudinal fiber axis. The line position was moved after each line-scan image. The majority of images were taken with a scanning frequency of either 1.15 ms or 1.92 ms per line. Image processing and analysis was performed using Image/J (NIH, USA) and Microcal Origin (Microcal Software Inc., Northampton, MA, USA).

### Ca^2+^ release calculation

Relative changes in rhod-2 fluorescence were expressed as *F/F_0_*, where *F_0_* is the resting (or baseline) fluorescence level. Changes in [Ca^2+^] were calculated from the rhod-2 signals using the previously described pseudo-ratio equation [25], assuming a basal [Ca^2+^] of 100 nM and a *K*_d_ of rhod-2 for Ca^2+^ of 1.2 µM. An estimation of the Ca^2+^ release flux underlying the calculated global [Ca^2+^] transients was performed according to a previously described procedures [26], [27]. Briefly, the SR calcium release flux was calculated from the time derivative of the total myoplasmic Ca^2+^ ([Ca]_T_) obtained from the occupancy of intracellular calcium binding sites. The model included troponin C binding sites with a total concentration of sites (*TN_total_*) of 250 µM, an “on” rate constant (*k_on, CaTN_*) of 0.0575 µM^−1^.ms^−1^ and an “off” rate constant (*k_off, CaTN_*) of 0.115 ms^−1^; Ca-Mg binding sites on parvalbumin with a total concentration of sites (*PV_total_* ) of 2000 µM, “on” rate constant for Ca^2+^ (*k_on, CaPV_*) of 0.125 µM^−1^.ms^−1^, “off” rate constant for Ca^2+^ (*k_off, CaPV_*) of 5.10^−4^ ms^−1^, “on” rate constant for Mg^2+^ (*k_on, MgPV_*) of 3.3.10^−5^ µM^−1^.ms^−1^, “off” rate constant for Mg^2+^ (*k_off, MgPV_* ) of 3.10^−3^ ms^−1^. Calcium transport across the SR membrane was included with a rate assumed to be proportional to the fractional occupancy of the SR pump sites with a dissociation constant (*Kd Ca_pump_*) of 2 µM and a maximum pump rate of 10 µM.ms^−1^. Resting [Mg^2+^] was assumed to be 1.5 mM. The model also included Ca^2+^-binding sites on EGTA at a concentration of 15 mM, an “on” rate constant (*k_on, CaEGTA_*) of 0.056 µM^−1^.ms^−1^ and an “off” rate constant (*k_off, CaEGTA_*) of 0.002 ms^−1^. Under the present recording conditions, calcium binding to EGTA made a predominant contribution to the calculated Ca^2+^ release flux as compared to the intrinsic Ca^2+^ -buffering and -removal components of the model. Analyses essentially focused on comparing the properties of Ca^2+^ transients and Ca^2+^ release within a given line-scan image between regions of the same fiber with and without detectable EGFP-RyR1 fluorescence.

### Electrophysiology

An RK-400 patch-clamp amplifier (Bio-Logic, Claix, France) was used in whole-cell voltage-clamp configuration. Fibers were bathed in a TEA-containing extracellular solution (see Voltage clamp solutions). Command voltage pulse generation and data acquisition were achieved with an A/D, D/A converter (Digidata 1440A, Molecular Devices, Sunnyvale, CA, USA) controlled by pClamp 9 software (Molecular Devices). Voltage-clamp was performed with the micropipette filled with a rhod-2 and EGTA containing intracellular solution (see Voltage clamp solutions). Analog compensation was systematically used to decrease the effective series resistance. Membrane depolarizing steps of 0.5 s duration were applied from a holding command potential of −80 mV.

### Immunofluorescence

*Fdb* and interosseus muscles were electroporated with the EGFP-G4896V RyR1 construct. Two weeks later, single fibers were isolated using collagenase treatment as described above. Muscles were triturated in Tyrode solution onto glass slides. The presence of EGFP-positive fibers on the slides was checked by fluorescence microscopy. Slides were air-dried and muscle fibers were fixed and permeabilized in cold methanol (−20°C) for 10 minutes. Slides were then stored at −80°C. Slides were re-hydrated in PBS buffer. RyR1 labeling was performed as described by Lefebvre et al. [20]. Briefly, slides were blocked for 50 min at room temperature (M.O.M. blocking kit, Vector Laboratories, BurlinGame, CA, USA) and then incubated overnight with a mouse anti-RyR monoclonal antibody (34C). Slides were washed for 5 min in PBS, incubated for 1 h with an anti-mouse Cy3-conjugated secondary antibody and then washed in PBS before being mounted. For DHPR and SERCA1 labeling, non-specific sites were first blocked for 50 min at room temperature with M.O.M. blocking kit (Vector Laboratories) and then with an Avidin/Biotin Blocking kit (Vector Laboratories). Cells were incubated overnight with a primary antibody either against DHPR α1s sub-unit (EMD Millipore, Billerica, MA, USA) or SERCA1 ATPase (ABR Affinity BioReagents, Golden, CO, USA), both raised in mouse, diluted at 1/250 and 1/500 in PBS buffer, respectively. Amplification was performed with the kit-biotinylated Anti-Mouse IgG Reagent and detection was made with streptavidin-labeled HilytePlus (ANASPEC, Fremont, CA, USA). Negative controls performed under identical conditions without primary antibody were fluorescence-free (see Fig. S1). Fluorescence was observed with a Zeiss LSM 5 confocal microscope (Carl Zeiss, Jena, Germany) and analyzed using ImageJ software (NIH, USA).

### Voltage clamp solutions

The intracellular voltage-clamp pipette solution contained (in mM) 120 K-glutamate, 5 Na_2_-ATP, 5 Na_2_-phosphocreatine, 5.5 MgCl_2_, 5 glucose, 0.1 rhod-2, 20 EGTA, 8 CaCl_2_ and 5 HEPES. The extracellular solution contained (in mM) 140 TEA-methanesulfonate, 2.5 CaCl_2_, 2 MgCl_2_, 10 TEA-HEPES and 0.002 tetrodotoxin. All solutions were adjusted to pH 7.20.

### Statistics

Least-squares fits were performed using a Marquardt-Levenberg algorithm routine included in Microcal Origin (Originlab, Northampton, MA, USA). Data values are presented as means ± SEM for *n* cells. Statistical significance was determined using a Student's t-test (One-Way Anova for data from cultured myotubes) with significance accepted at P<0.05 (*) and P<0.01 (**).

## Results

### Functional analysis of G4896V and R4892W RyR1 channels in dyspedic myotubes

**Figure 1:**
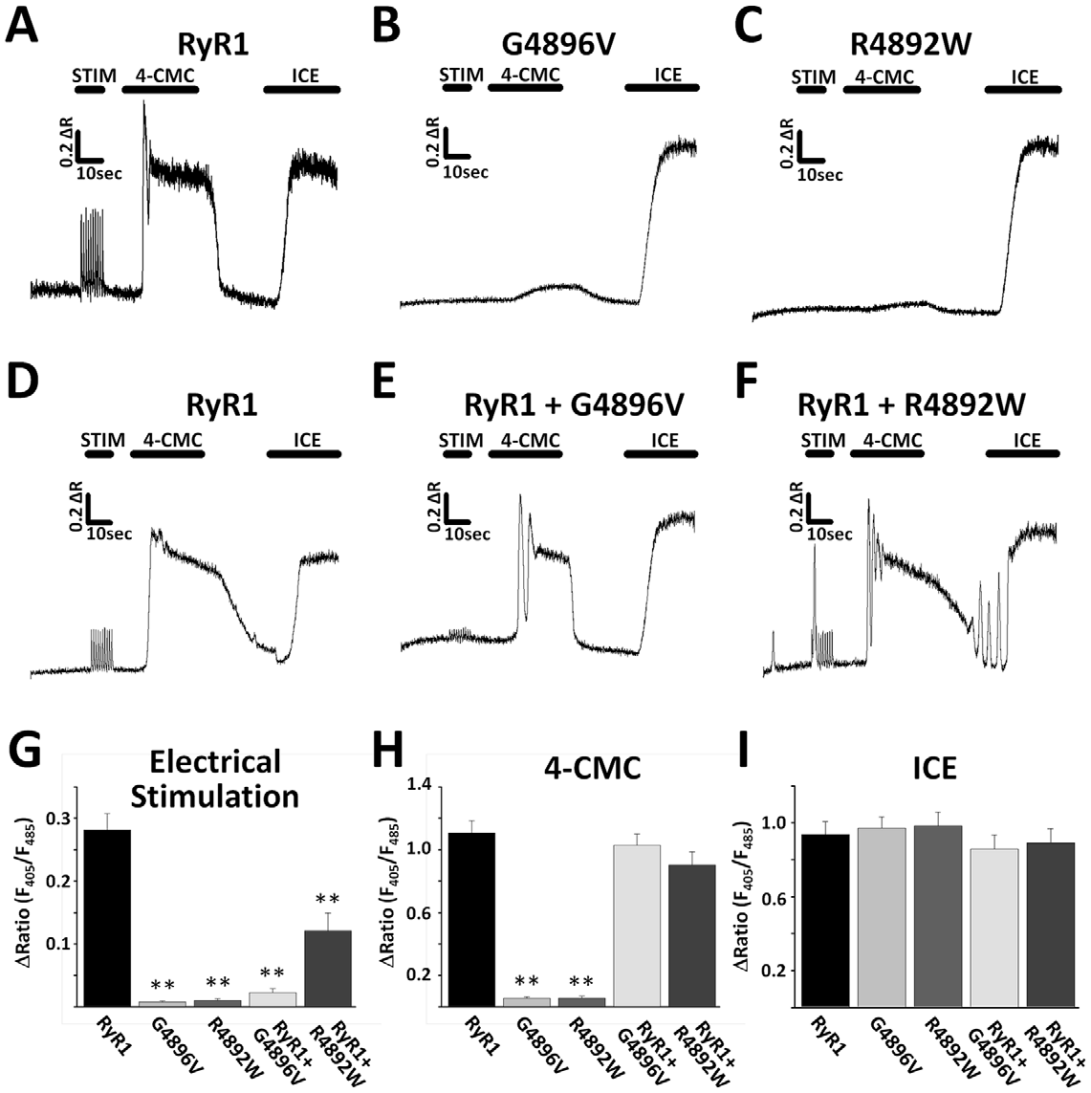
Effects of the G4896V and R4892W CCD mutations in RyR1 on electrically-evoked and ligand-induced Ca^2+^ release in dyspedic myotubes. *A–C*, representative Ca^2+^ measurements from intact indo-1-loaded dyspedic myotubes following homotypic expression of either wild-type (WT) RyR1 (*A*), G4896V (*B*), or R4892W (*C*). *D–F*, representative Ca^2+^ measurements from intact indo-1-loaded dyspedic myotubes following heterotypic expression of either WT RyR1 alone (*D*), WT + G4896V (*E*), or WT + R4892W (*F*). *G–I*, bar graphs summarizing the average (± S.E.M.) peak magnitude of Ca^2+^ release induced by electrical stimulation (*G*), addition of 500 µM 4-CMC (*H*), or application of an intracellular Ca^2+^ release cocktail (ICE – 10 µM ionomycin, 30 µM CPA, and 100 µM EGTA/0 Ca^2+^ Ringer's solution) (*I*). Results in panels *G–I* are from 19, 15, 12 and 15 G4896V, R4892W, G4896V/WT and R4892W/WT RyR1-expressing cells, respectively, and from 18 (*G*), 16 (*H*) and 15 (*I*) WT-RyR1 expressing cells.

Homotypic expression in dyspedic myotubes of RyR1 channels with CCD mutations located in the selectivity filter and adjacent pore lining region markedly reduces the magnitude of voltage- and ligand induced Ca^2+^ release in a manner that is independent of a detectable change in total SR Ca^2+^ content, a functional phenotype referred to as “EC uncoupling” [8], [9]. Fig. 1A–C shows representative traces from indo-1-loaded dyspedic myotubes following homotypic expression of either wild-type (WT) RyR1, a CCD mutation directly within the RyR1 selectivity filter (G4896V), or a CCD mutation immediately adjacent to the pore lining region of RyR1 (R4892W). For each homotypic expression condition, we quantified electrically-evoked (1 Hz for 10 seconds) Ca^2+^ release, maximal 4-chloro-m-cresol-induced (500 µM) Ca^2+^ release, and total intracellular Ca^2+^ store content following addition of a Ca^2+^ release cocktail (ICE) consisting of 10 µM ionomycin and 30 µM cyclopiazonic acid in a Ca^2+^-free (100 µM EGTA/0Ca^2+^) Ringer's solution. WT RyR1-expressing myotubes exhibit robust responses to electrical stimulation, 4-CMC application, and ICE-induced liberation of Ca^2+^ stores (Fig. 1A). However, while G4896V- (Fig. 1B) and R4892W-expressing (Fig. 1C) myotubes lack electrically-evoked and 4-CMC-induced Ca^2+^ release, Ca^2+^ store content is comparable to that of WT RyR1-expressing myotubes. Thus, G4896V- and R4892W-expressing myotubes exhibit a classic EC uncoupling phenotype.

Since CCD is an autosomal dominant disorder and patients are heterozygous for the G4896V and R4892W mutations, we also conducted similar experiments following heterotypic expression of each mutant at a 1∶1 ratio with WT RyR1 (Fig. 1D–F). Compared to homotypic expression, co-expression of each mutant together with WT RyR1 partially rescued electrically-evoked Ca^2+^, though a significantly greater increase was observed for heterotypic expression of R4892W (Fig. 1G). Maximal 4-CMC-induced Ca^2+^ release and total Ca^2+^ store content were not significantly different for any of the expression conditions (Fig. 1H and I), though the maximum rate of rise in indo-1 ratio upon 4-CMC addition was significantly slower following heterotypic expression of G4896V (data not shown). These results demonstrate that both homotypic and heterotypic expression of the G4896V and R4892W CCD mutations in RyR1 in dyspedic myotubes result in EC uncoupling.

### Expression of exogenous G4896V and R4892W RyR1 channels in adult muscle fibers

**Figure 2:**
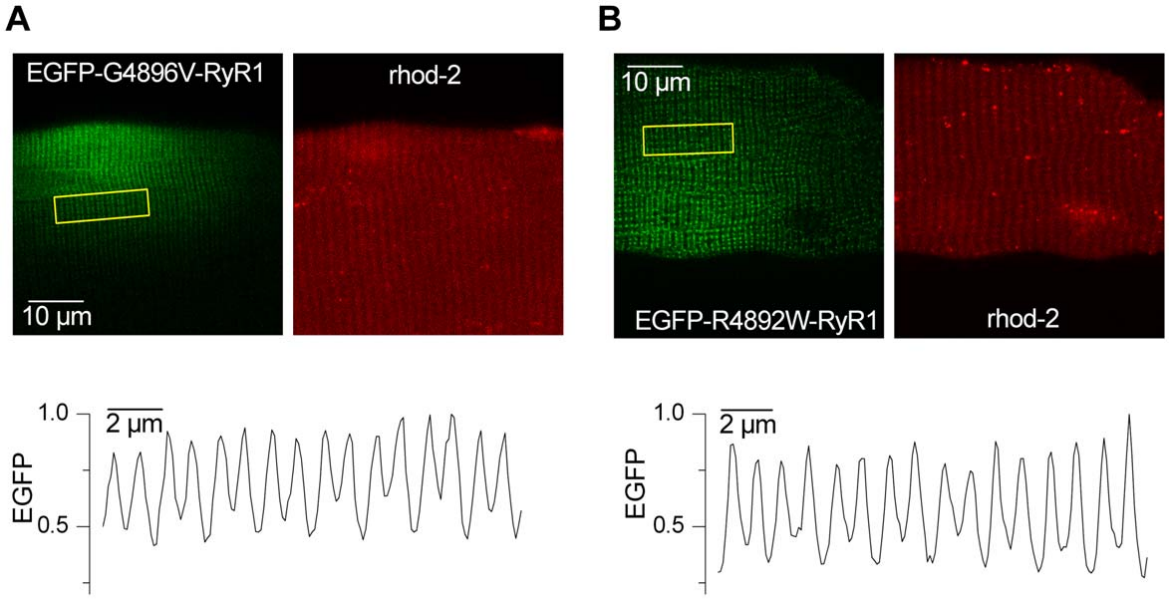
Pattern of EGFP-tagged G4896V and R4892W mutant RyR1 channel expression in adult mouse muscle fibers. *A*, representative example of the expression pattern of EGFP-G4896V (*left*) and of the corresponding rhod-2 fluorescence (*right*) in the same fiber. *B*, representative example of the expression pattern of EGFP-R4892W (*left*) and of the corresponding rhod-2 fluorescence (*right*) in the same fiber. In *A* and *B* the graph below each pair of images shows the relative fluorescence intensity profile for EGFP (values were divided by the maximum) within the boxed region in the corresponding EGFP image.

We next set out to determine the effects of the G4896V and R4892W mutants on voltage-gated Ca^2+^ release in fully-differentiated adult skeletal muscle fibers. For these experiments, N-terminal EGFP-tagged versions of G4896V and R4892W RyR1 were transiently expressed in *fdb* and interosseus muscles of 6–8 week-old WT mice. Figure 2 shows illustrative examples of the localized expression patterns observed for EGFP-G4896V and EGFP-R4892W RyR1channels. As observed previously for other RyRs exogenously expressed in adult muscle [19], [20], both CCD mutant channels were expressed within spatially restricted regions of transfected fibers (see Fig. S1) where they exhibited a transversal double-banded pattern with a characteristic ∼2 µm spacing, consistent with likely localization within the terminal cisternae region of the SR. There was no particular feature that could distinguish the expression pattern of one mutant from the other one in terms of profile, spatial extent or, although qualitative, EGFP intensity. In addition, the magnitude of the rhod-2 signal was consistent both within and outside regions of EGFP fluorescence, indicating the absence of large local gradients in myoplasmic Ca^2+^. Finally, although no systematic quantification was made, both mutants were found to express in adult fibers to a similar extent and frequency.

### Properties of SR Ca^2+^ release in fiber regions endowed with EGFP-G4896V channels

**Figure 3:**
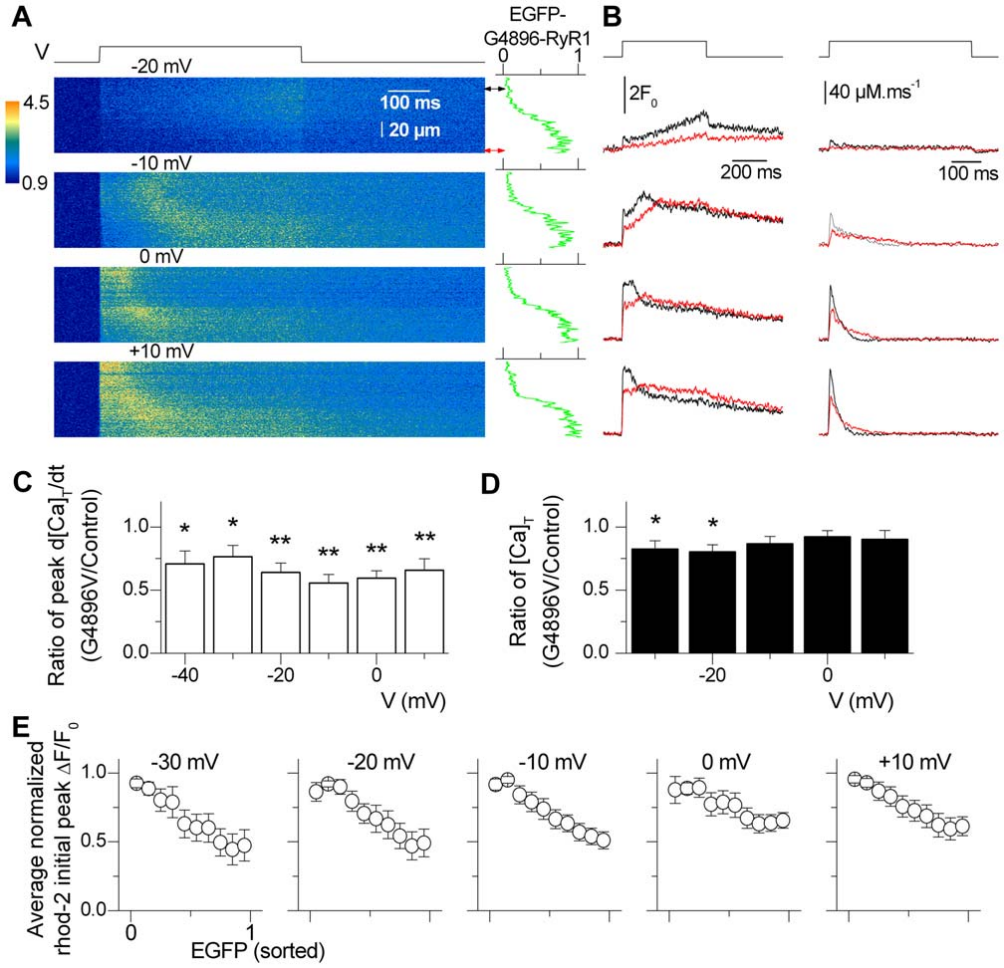
Voltage-activated SR Ca^2+^ release in a fiber expressing EGFP-G4896V. *A*, representative confocal (*x,t*) line-scan images of rhod-2 fluorescence taken from a region of a muscle fiber that included an area of high localized EGFP-G4896V expression. The fiber was depolarized by 0.5 s-long test pulses from −80 mV to the indicated potentials. The graph on the right of each image shows the EGFP fluorescence intensity profile along the line. The double-arrows indicate regions of low and high expression from which the traces shown in *B* were calculated. *B*, time course of relative (*F/F_0_*) rhod-2 fluorescence (*left*) and of the corresponding calculated Ca^2+^ release flux (*right*) for the two regions indicated by double-arrows in *A*. The black and red traces were calculated from regions of low and high expression, respectively. *C*, voltage-dependence of the average (± S.E.M.) ratio of peak Ca^2+^ release measured in regions of high EGFP-G4896V expression relative to that observed in regions of low expression along the same line-scan image. *D*, voltage-dependence of the average (± S.E.M.) ratio of total released Ca^2+^ corresponding to the data shown in *C*. Data are from 8 fibers. *E*, dependence of the initial average (± S.E.M.) peak Ca^2+^ transient amplitude on relative EGFP intensity for the different test depolarizations. Initial peak *F/F_0_* was taken at each *x* location along the scan line as the average of consecutive values near the peak over a time interval of 20 ms. Initial peak *F/F_0_* values were averaged according to relative EGFP signal intensity values binned at 0.1.

We previously reported that the control of voltage-gated SR Ca^2+^ release is well maintained even in regions of adult fibers exhibiting forced exogenous expression of EGFP-WT RyR1 channels [20]. Fig. 3A shows a series of line-scan images of rhod-2 fluorescence from a fiber expressing EGFP-G4896V. The line position for these images was set throughout a fiber region where the EGFP signal yielded a broad intensity gradient: the EGFP profile along the scanned line is shown on the right side of each image. During the line-scan, a 500 ms duration voltage-clamp depolarization from −80 mV to the indicated value was applied. The left series of traces shown in Fig. 3B compares the normalized rhod-2 fluorescence (F/F_0_) calculated at the two line positions where the EGFP signal was maximal and minimal, respectively (positions are indicated by double-arrows next to the uppermost line-scan image). The rhod-2 signal was averaged over 50 adjacent rows. The right series of traces in Fig. 3B shows the corresponding Ca^2+^ release flux. For both the F/F_0_ and Ca^2+^ release flux, traces from the EGFP-positive region of the fiber are displayed in red. For all values of membrane depolarization, the initial peak of the rhod-2 fluorescence transient and of the corresponding Ca^2+^ release flux were reduced within the EGFP positive region of the fiber. Importantly, as observed previously [20], as peak Ca^2+^ release increased with stronger depolarizing pulses, its late decay phase was faster, consistent with SR Ca^2+^ depletion occurring early during depolarization as peak Ca^2+^ release increased. As described previously [20], we made the simplifying approximation that any severe change in SR Ca^2+^ content due to expression of mutant RyR1s should be reflected as a decrease in the value of total released Ca^2+^, estimated from the time integral of the Ca^2+^ release flux during strong depolarizing pulses. In Fig. 3B, the lower peak Ca^2+^ release in the high-expression region of the fiber (indicated by the red double-arrow in Fig. 3A) qualitatively correlated well with a slower decay of the Ca^2+^ flux, indicating that a lower initial SR content was likely not the reason for the reduced peak value. For statistical comparison of the data between the two fiber regions with differing EGFP levels, we calculated the ratio of peak Ca^2+^ release and of total released Ca^2+^ in the region of largest expression of EGFP-G4896V relative to the corresponding value in the region of lowest expression, within the same line-scan image. The ratio should equal unity if the value for peak Ca^2+^ release (or total released Ca^2+^) is identical in the two regions of the line-scan. Figure 3C shows the voltage dependence of the mean value of this ratio for peak Ca^2+^ release flux in fibers expressing EGFP-G4896V. Statistical significance was tested against the hypothesis of the ratio being equal to unity. Throughout the voltage range tested (−40 to +10 mV), all values were significantly lower than unity, with an average corresponding to an ∼30% decrease in peak Ca^2+^ release flux. Conversely, the corresponding mean ratio for total released Ca^2+^ yielded values that statistically did not differ from 1 for the largest levels of depolarization (Fig. 3D), consistent with the absence of a change in SR Ca^2+^ content between fiber regions expressing high and low levels of EGFP-G4896V channels.

Figure 3E shows the result from a spatial correlation between the peak amplitude of the rhod-2 Ca^2+^ transient and EGFP expression: it shows the relationship between the mean relative level of EGFP intensity and the mean relative change in peak rhod-2 fluorescence (normalized ΔF/F_0_) along the lines scanned in EGFP-G4896V-expressing fibers, for the different test potentials. For each image, the initial peak ΔF/F_0_ value in response to the pulse was calculated at each *x* position along the line: in order to reduce the noise, the average of consecutive values over a 20 ms time interval centered around the time of the peak of the global transient was used in the calculation. Pairs of ΔF/F_0_-EGFP values were then sorted and averaged according to values falling within a given range of EGFP amplitudes with an increment arbitrarily set to 0.1 unit of relative EGFP signal, as described by Lefebvre et al. [20]. For each image, the resulting set of 10 peak ΔF/F_0_ values was then divided by the largest one to give the normalized ΔF/F_0_ values. These sets of normalized values from the different fibers were then averaged for each membrane potential tested. For all test depolarizations, a clear negative correlation was found between EGFP expression and peak Ca^2+^ transient amplitude, with a similar negative slope observed for each membrane potential tested.

### Properties of SR Ca^2+^ release in fiber regions endowed with EGFP-R4892W channels

**Figure 4:**
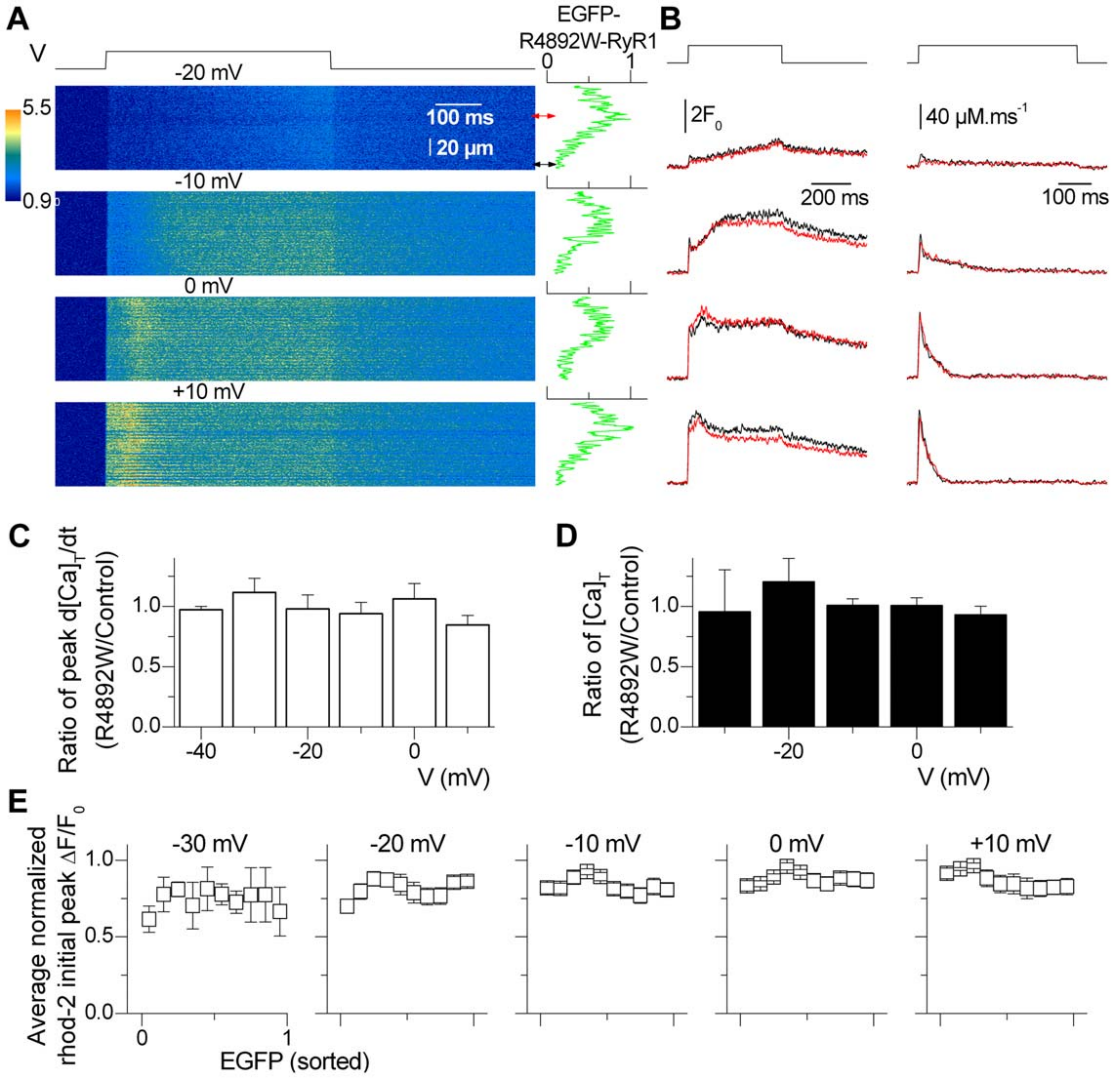
Voltage-activated SR Ca^2+^ release in a fiber expressing EGFP-R4892W. *A*, representative confocal (*x,t*) line-scan images of rhod-2 fluorescence taken from a region of a muscle fiber that included an area of high localized EGFP-R4892W expression. The fiber was depolarized by 0.5 s-long test pulses from −80 mV to the indicated potentials. The graph on the right of each image shows the EGFP fluorescence intensity profile along the line. The double-arrows indicate regions of low and high expression from which the traces shown in *B* were calculated. *B*, time course of relative (*F/F_0_*) rhod-2 fluorescence (*left*) and of the corresponding calculated Ca^2+^ release flux (*right*) for the two regions indicated by double-arrows in *A*. The black and red traces were calculated from regions of low and high expression, respectively. *C*, voltage-dependence of the average (± S.E.M.) ratio of peak Ca^2+^ release measured in regions of high EGFP-R4892W expression relative to that observed in regions of low expression along the same line-scan image. *D*, corresponding voltage-dependence of the average (± S.E.M.) ratio of total released Ca^2+^ corresponding to the data shown in *C*. Data are from 8 fibers. *E*, dependence of the initial average (± S.E.M.) peak Ca^2+^ transient on relative EGFP intensity for the different test depolarizations. Initial peak *F/F_0_* was taken at each *x* location along the scan line as the average of consecutive values near the peak over a time interval of 20 ms. Initial peak *F/F_0_* values were averaged according to the relative EGFP signal intensity values binned at 0.1.

Figure 4 summarizes results obtained from EGFP-R4892W-expressing fibers using the same format as Figure 3. Figure 4A shows line-scan images of rhod-2 fluorescence from a representative fiber expressing EGFP-R4892W channels, with the EGFP profile along the scanned line shown on the right side of each image. The left series of traces shown in Fig. 4B compares the normalized rhod-2 fluorescence (F/F_0_) calculated within high (red traces) and low (black traces) EGFP-R4892W-expressing regions of the line, whereas the series of traces on the right show the corresponding Ca^2+^ release flux. Rhod-2 F/F_0_ transients and corresponding Ca^2+^ release flux traces were very similar in the two regions.

Figure 4C–D shows the voltage dependence of the mean ratio for peak Ca^2+^ release and for total released Ca^2+^, respectively, in regions of high and low EGFP-R4892W expression. In contrast with that observed for EGFP-G4896V, a significant change in Ca^2+^ release was not observed at any test potential in areas high in EGFP-R4892W expression (Fig. 4C). This finding was also confirmed by the absence of a correlation between the relative peak initial rhod-2 fluorescence (F/F_0_) and the corresponding EGFP signal (Fig. 4E).

### Cav1.1 and SERCA1 localization is unaltered in GFP-G4896V-expressing fibers

**Figure 5:**
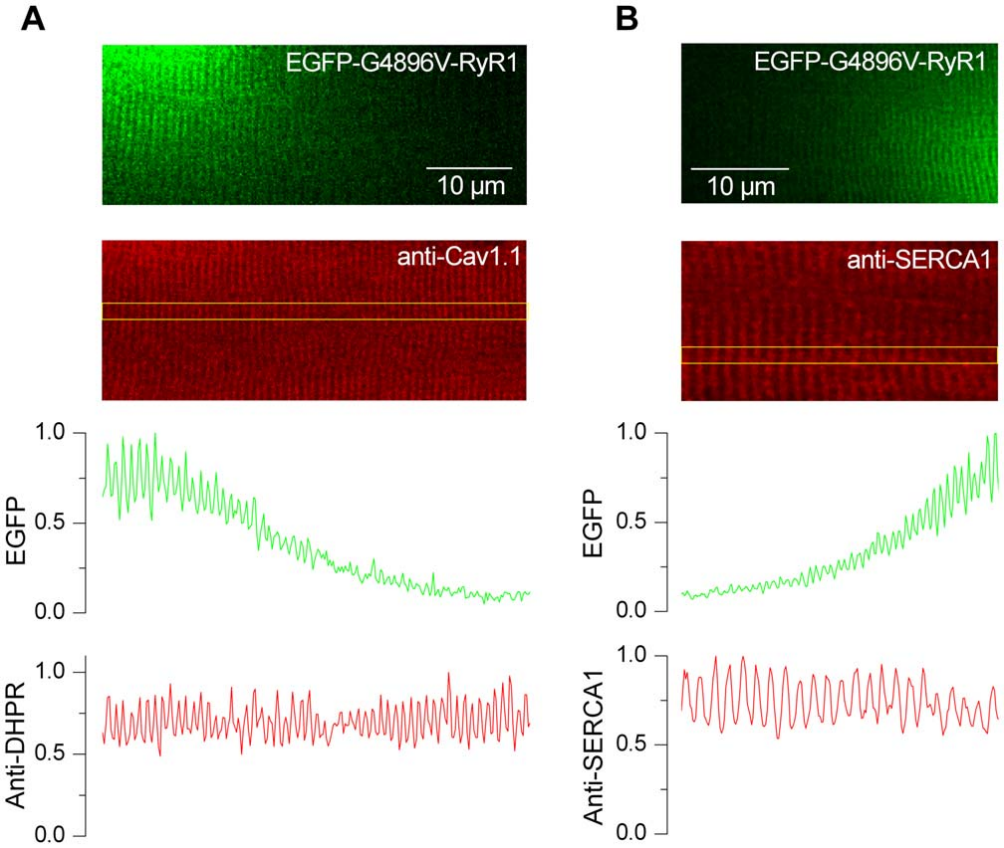
Immunofluorescence labeling of DHPR and SERCA1 in fibers expressing EGFP-G4896V. *A*, representative confocal (*x,y*) image of EGFP fluorescence (*top image*) and DHPR immunofluorescence (*lower image*) from an EGFP-G4896V-expressing fiber. EGFP (*upper graph*) and DHPR (*lower graph*) fluorescence intensity profiles along the boxed region shown in the DHPR immunofluorescence image. *B*, representative confocal (*x,y*) image of EGFP fluorescence (*top image*) and SERCA1 immunofluorescence (*lower image*) from an EGFP-G4896V-expressing fiber. EGFP (*upper graph*) and SERCA1 (*lower graph*) fluorescence intensity profiles along the boxed region shown in the SERCA1 immunofluorescence image.

Using muscle fibers transfected with the same approach that was used here, we previously reported that overall RyR1 channel density remained homogeneous even across fiber regions exogenously expressing CCD-associated EGFP-I4897T mutant channels [20]. A similar uniform total RyR1 expression was also observed in EGFP-G4896V- and EGFP-R4892W-expressing fibers: there was no correlation between EGFP intensity gradient and total RyR1 antibody signal (data not shown). In addition, we also determined the potential impact of exogenous mutant RyR1 expression on the relative expression and localization of other junctional proteins critically involved in Ca^2+^ homeostasis and EC coupling. Specifically, we performed immunostaining of both the DHPR α_1S_ subunit (Fig. 5A) and SERCA1 (Fig. 5B) in EGFP-G4896V-expressing fibers. In this context, it is worth mentioning that any attempt of quantifying the DHPR functional expression from membrane current measurements would be limited by the variable extent of local fiber volume occupied by expressed mutant channels from one fiber to another and was thus considered unreliable. The top frame in Fig. 5A shows the fluorescence from expressed EGFP-G4896V channels and the frame below shows the corresponding signal measured using an antibody directed against the α_1S_ subunit of the DHPR. The two line graphs on the bottom panel of Fig. 5A show the EGFP-G4896V (*upper*) and DHPR (*lower*) fluorescence profiles along the box regions highlighted in the anti-DHPR frame. Whereas the EGFP signal almost completely vanished from left to right, the typical periodic DHPR signal intensity remained constant across the same region. Similar results were observed in 4 separate fibers. Parallel experiments using a SERCA1 antibody revealed a similar constant periodic SERCA1 signal intensity across a region of the fiber exhibiting a strong gradient of EGFP-G4896V expression (Fig. 5B). Similar results were observed in 5 muscle fibers. The spatial frequency of the transversal SERCA1 banded pattern was approximately half that of DHPR and EGFP, as expected since SERCA1 is located primarily in the longitudinal SR within the Z-disk region of the sarcomere, whereas DHPR and RyR1 proteins are located in the two triadic regions at the A-I band junction of the sarcomere in mammalian skeletal muscle. Although localized expression of EGFP-R4892W did not significantly alter Ca^2+^ release in adult muscle fibers (Fig. 4), periodic DHPR and SERCA1 signal intensities were also unaffected by localized expression of EGFP-R4892W channels (data not shown).

## Discussion

The human equivalents of the N-terminal EGFP-tagged G4896V and R4892W mutations in rabbit RyR1 characterized here are both associated with CCD. Our results demonstrate that these two RyR1 mutants exhibit different functional outcomes on voltage-activated SR Ca^2+^ release when present with WT RyR1 in either dyspedic myotubes or fully differentiated skeletal muscle fibers of adult mice. Together with our previous studies [20], these results demonstrate a heterogeneity of functional consequences at the cellular level attributable to CCD-associated mutations in RyR1 when transiently expressed within a skeletal muscle environment. This heterogeneity is likely to contribute, at least in part, to the variable presentation, progression and penetrance of symptoms in CCD patients.

The human equivalent of the G4896V mutation in rabbit RyR1 was linked to 8 individuals diagnosed with CCD in the same family [28]. However, functional analysis of this putative RyR1 CCD variant has so far never been studied in any expression system. We demonstrate here for the first time that homotypic G4896V channels expressed in dyspedic myotubes are EC uncoupled and exert a potent dominant-negative suppression of voltage-gated Ca^2+^ release when coexpressed with WT RyR1 channels (Fig. 1). Consistent with these results, dominant-negative suppression of voltage-gated Ca^2+^ release in the absence of a change in SR Ca^2+^ content was also observed following transient expression of N-terminally-tagged EGFP-G4896V RyR1 channels in *fdb* fibers of adult WT mice.

A recent study reported that expression of CCD- and MHS-associated RyR1 mutants in C2C12 myotubes resulted in reciprocal changes in SERCA2 Ca^2+^-pump gene expression [29]. Compensatory changes in proteins involved in controlling SR Ca^2+^ release or reuptake could significantly impact the myoplasmic Ca^2+^ transient during EC coupling. However, we found that exogenous expression of EGFP-G4896V in adult muscle fibers did not significantly alter the subcellular localization or relative expression of either SERCA1 or DHPR. In addition, EGFP-G4896V did not result in a detectable change in SR Ca^2+^ content. Thus, the reduction in depolarization-induced Ca^2+^ release in adult fibers following exogenous expression of GFP-G4896V most likely reflects direct effects of this CCD mutant on RyR1 Ca^2+^ release.

Interestingly, the G4896V mutant results in a mutation to the first glycine residue in the highly-conserved RyR1 “GIG” selectivity filter [30]–[33] immediately preceding an isoleucine residue, of which mutation to a threonine is the most common CCD mutation in humans. In fact, our results here with G4896V are qualitatively similar to those we reported previously for I4897T expressed in adult muscle fibers under identical conditions [20]. Specifically, we found that expression of the EGFP-G4896V mutant in fully-differentiated adult muscle fibers of WT mice reduced voltage-gated Ca^2+^ release by ∼30%, which was somewhat less than the ∼50% reduction observed previously for the I4897T mutant [20]. This similarity is not unexpected since the “GIG” motif is a critical determinant of RyR1 ion selectivity and conduction [34] and CCD mutations to these residues reduce RyR1 channel conductance and Ca^2+^ permeation [18], [32], [35].

Assuming the G4896V and I4897T mutant monomers yield similar defects in tetrameric RyR1 channel function, the difference in magnitude of suppression in voltage-gated Ca^2+^ release observed for the G4896V and I4897T mutants could reflect a lower degree of replacement of endogenous WT RyR1 monomers by G4896V. For example, using the single channel data from Loy et al. [18] relating RyR1 Ca^2+^ conduction to the number of mutant I4897T monomers incorporated into a tetrameric channel, the 50% and 30% reduction in Ca^2+^ release observed in our experiments would correspond to a probability of replacement of endogenous monomers by mutants that would not exceed 0.6 and 0.45 for I4897T and G4896V, respectively. Alternatively, the difference may be due to the G4896V mutant exhibiting a similar probability of replacement but producing a more modest alteration in release channel function.

CCD-linked mutations at positions I4897 (I4897T) and G4898 (G4898E/R) do not support voltage-gated Ca^2+^ release upon homotypic expression in dyspedic myotubes [9]. In addition, previous studies of recombinant homotypic channels incorporated into lipid bilayers demonstrated that these CCD mutations markedly reduce RyR1 channel conduction and eliminate Ca^2+^ ion permeation [18], [35]. Similar effects were previously reported for other non-disease related amino acid substitutions of I4897 and G4898 [31], [32]. The current study is the first to determine the impact on RyR1 function of a disease mutation of the first glycine residue in the critical “GIG” selectivity filter. Our results demonstrate that the mutation abolished Ca^2+^ release when expressed alone and imparts a strong dominant-negative inhibition of release when co-expressed with WT RyR1. The relative impact of different mutations at position G4896 in comparison to I4897 and G4898 on RyR1 channel conductance and permeation will require systematic single channel analyses. Nevertheless, our findings provide compelling evidence that expression of G4896V monomers is sufficient to inhibit voltage-gated SR Ca^2+^ release in both myotubes and differentiated muscle fibers as was demonstrated previously for the I4897T mutant [8], [18], [20].

The human equivalent of the R4892W mutation was first identified in individuals from two unrelated families [36]. Homotypic expression of the R4892W mutant failed to support either ligand- or voltage-gated Ca^2+^ release (EC uncoupled) when expressed in dyspedic myotubes (9; Fig. 1A and G) or either caffeine- or Ca^2+^-induced Ca^2+^ release in HEK-293 cells [37]. Interestingly, in contrast with other CCD-associated mutants including I4897T, significant yet brief caffeine responses were observed in R4892W-expressing dyspedic myotubes, indicative of a somewhat maintained Ca^2+^ sensitivity in muscle cells [9]. In addition, when expressed in HEK-293 cells together with WT RyR1 in a 1∶2 ratio, Ca^2+^ sensitivity of release was fully restored for R4892W, while only partially restored for I4897T and G4898E [37].

Here, we found that the dominant-negative effect of the R4892W mutant on voltage-gated Ca^2+^ release in myotubes and adult muscle fibers was considerably less than that observed for G4896V. This difference may in part be due to the fact that residues I4897 and G4896 are core components of the RyR1 selectivity filter, whereas R4892 is most likely only indirectly involved [34]. Still, the fact that homotypic expression of R4892W fails to support voltage-gated Ca^2+^ release in the dyspedic myotubes (as is the case for G4896V and I4897T RyR1s) explicitly demonstrates a defective function of these channels, consistent with the muscle weakness observed in patients. Thus, the observation that (unlike G4896V and I4897T) a Ca^2+^ release defect was not observed following expression of R4892W in adult muscle was unexpected. Assuming that the different mutant channels are produced, processed and targeted in the fibers with a similar efficiency, the lack of defective Ca^2+^ release associated with R4892W expression in adult muscle could reflect either a specific requirement for incorporation of more mutant monomers into a hetero-tetrameric channel or a compensatory effect due to a maintained Ca^2+^-sensitivity of the heterotypic channels [9], [37]. Whatever the exact reason, these findings indicate that differences with regard to relative mutant RyR1 expression, tetramer incorporation, and potency of dominant-negative suppression of release channel function are likely contribute to the high phenotypic variability and low penetrance that is characteristic of CCD in humans.

## Supporting Information

Figure S1 **Immunofluorescence labeling of SERCA1 in fibers expressing EGFP-G4896V and EGFP-R4892W mutant RyR1 channels.** Examples of local expression of either EGFP-G4896V-RyR1 (top and bottom left panels) or EGFP-R4892W-RyR1 (medium left panel) and the corresponding SERCA1 labeling. The bottom right image shows a negative control image obtained in the absence of primary antibody.
(TIF)

## References

[1] Lyfenko AD, Goonasekera SA, Dirksen RT (2004) Dynamic alterations in myoplasmic Ca^2+^ in malignant hyperthermia and central core disease. Biochem Biophys Res Commun 322: 1256–1266. 15336973 10.1016/j.bbrc.2004.08.031

[2] Treves S, Anderson AA, Ducreux S, Divet A, Bleunven C, et al (2005) Ryanodine receptor 1 mutations, dysregulation of calcium homeostasis and neuromuscular disorders. Neuromuscul Disord 15: 577–587. 16084090 10.1016/j.nmd.2005.06.008

[3] Treves S, Jungbluth H, Muntoni F, Zorzato F (2008) Congenital muscle disorders with cores: the ryanodine receptor calcium channel paradigm. Curr Opin Pharmacol 8: 319–326. 18313359 10.1016/j.coph.2008.01.005

[4] Rosenberg H, Sambuughin N, Dirksen R (2010) Malignant hyperthermia susceptibility. In Gene Reviews^TM^[Internet]. R.A. Pagon, T.D. Bird, C.R. Dolan, K. Stephens, and M.P. Adams, editors. University of Washington, Seattle.

[5] Muntoni F, Sewry CA (2003) Central core disease: new findings in an old disease. Brain 126: 2339–2340. 14561637 10.1093/brain/awg288

[6] Robinson R, Carpenter D, Shaw MA, Halsall J, Hopkins P (2006) Mutations in RYR1 in malignant hyperthermia and central core disease. Hum Mutat 27: 977–989. 16917943 10.1002/humu.20356

[7] Jungbluth H (2007) Central core disease. Orphanet J Rare Dis 2: 25. 17504518 10.1186/1750-1172-2-25 PMC1887524

[8] Avila G, O'Brien JJ, Dirksen RT (2001) Excitation-contraction uncoupling by a human central core disease mutation in the ryanodine receptor. Proc Natl Acad Sci USA 98: 4215–4220. 11274444 10.1073/pnas.071048198 PMC31205

[9] Avila G, O'Connell KM, Dirksen RT (2003) The pore region of the skeletal muscle ryanodine receptor is a primary locus for excitation-contraction uncoupling in central core disease. J Gen Physiol 121: 277–286. 12642598 10.1085/jgp.200308791 PMC2217374

[10] Dirksen RT, Avila G (2004) Distinct effects on Ca^2+^ handling caused by malignant hyperthermia and central core disease mutations in RyR1. Biophys J 87: 3193–3204. 15347586 10.1529/biophysj.104.048447 PMC1304789

[11] Lynch PJ, Tong J, Lehane M, Mallet A, Giblin L, et al (1999) A mutation in the transmembrane/luminal domain of the ryanodine receptor is associated with abnormal Ca^2+^ release channel function and severe central core disease. Proc Natl Acad Sci USA 30: 4164–4169. 10.1073/pnas.96.7.4164 PMC22438 10097181

[12] Ducreux S, Zorzato F, Müller C, Sewry C, Muntoni F, et al (2004) Effect of ryanodine receptor mutations on interleukin-6 release and intracellular calcium homeostasis in human myotubes from malignant hyperthermia-susceptible individuals and patients affected by central core disease. J Biol Chem 279: 43838–43846. 15299003 10.1074/jbc.M403612200

[13] Chelu MG, Goonasekera SA, Durham WJ, Tang W, Lueck JD, et al (2006) Heat- and anesthesia-induced malignant hyperthermia in an RyR1 knock-in mouse. FASEB J 20: 329–330. 16284304 10.1096/fj.05-4497fje

[14] Boncompagni S, Rossi AE, Micaroni M, Hamilton SL, Dirksen RT, et al (2009) Characterization and temporal development of cores in a mouse model of malignant hyperthermia. Proc Natl Acad Sci U S A 106: 21996–22001. 19966218 10.1073/pnas.0911496106 PMC2799858

[15] Zvaritch E, Depreux F, Kraeva N, Loy RE, Goonasekera SA, et al (2007) An Ryr1I4895T mutation abolishes Ca^2+^ release channel function and delays development in homozygous offspring of a mutant mouse line. Proc Natl Acad Sci USA 104: 18537–18542. 18003898 10.1073/pnas.0709312104 PMC2141812

[16] Zvaritch E, Kraeva N, Bombardier E, McCloy RA, Depreux F, et al (2009) Ca^2+^ dysregulation in Ryr1(I4895T/wt) mice causes congenital myopathy with progressive formation of minicores, cores, and nemaline rods. Proc Natl Acad Sci USA 106: 21813–21818. 19959667 10.1073/pnas.0912126106 PMC2788482

[17] Boncompagni S, Loy RE, Dirksen RT, Franzini-Armstrong C (2010) The I4895T mutation in the type 1 ryanodine receptor induces fiber-type specific alterations in skeletal muscle that mimic premature aging. Aging Cell 9: 958–970. 20961389 10.1111/j.1474-9726.2010.00623.x PMC2980556

[18] Loy RE, Orynbayev M, Xu L, Andronache Z, Apostol S, et al (2011) Muscle weakness in Ryr1I4895T/WT knock-in mice as a result of reduced ryanodine receptor Ca^2+^ ion permeation and release from the sarcoplasmic reticulum. J Gen Physiol 137: 43–57. 21149547 10.1085/jgp.201010523 PMC3010056

[19] Legrand C, Giacomello E, Berthier C, Allard B, Sorrentino V, et al (2008) Spontaneous and voltage-activated Ca^2+^ release in adult mouse skeletal muscle fibres expressing the type 3 ryanodine receptor. J Physiol 586: 441–457. 18006577 10.1113/jphysiol.2007.145862 PMC2375597

[20] Lefebvre R, Legrand C, González-Rodríguez E, Groom L, Dirksen RT, et al (2011) Defects in Ca^2+^ release associated with local expression of pathological ryanodine receptors in mouse muscle fibres. J Physiol 589: 5361–5382. 21969454 10.1113/jphysiol.2011.216408 PMC3240878

[21] Avila G, Dirksen RT (2001) Functional effects of central core disease mutations in the cytoplasmic region of the skeletal muscle ryanodine receptor. J Gen Physiol 118: 277–290. 11524458 10.1085/jgp.118.3.277 PMC2229502

[22] Kimura T, Lueck JD, Harvey PJ, Pace SM, Ikemoto N, et al (2009) Alternative splicing of RyR1 alters the efficacy of skeletal EC coupling. Cell Calcium 45: 264–274. 19131108 10.1016/j.ceca.2008.11.005 PMC2743929

[23] Weiss N, Legrand C, Pouvreau S, Bichraoui H, Allard B, et al (2010) In vivo expression of G-protein beta1gamma2 dimer in adult mouse skeletal muscle alters L-type calcium current and excitation-contraction coupling. J Physiol 588: 2945–2960. 20547679 10.1113/jphysiol.2010.191593 PMC2956909

[24] Jacquemond V (1997) Indo-1 fluorescence signals elicited by membrane depolarization in enzymatically isolated mouse skeletal muscle fibers. Biophys J 73: 920–928. 9251808 10.1016/S0006-3495(97)78124-4 PMC1180988

[25] Cheng H, Lederer WJ, Cannell MB (1993) Calcium sparks: elementary events underlying excitation-contraction coupling in heart muscle. Science 262: 740–744. 8235594 10.1126/science.8235594

[26] Collet C, Pouvreau S, Csernoch L, Allard B, Jacquemond V (2004) Calcium signaling in isolated skeletal muscle fibers investigated under “Silicone Voltage-Clamp” conditions. Cell Biochem Biophys 40: 225–236. 15054224 10.1385/CBB:40:2:225

[27] Pouvreau S, Csernoch L, Allard B, Sabatier JM, De Waard M, et al (2006) Transient loss of voltage control of Ca^2+^ release in the presence of maurocalcine in skeletal muscle. Biophys J 91: 2206–2215. 16782801 10.1529/biophysj.105.078089 PMC1557560

[28] Kossugue PM, Paim JF, Navarro MM, Silva HC, Pavanello RC, et al (2007) Central core disease due to recessive mutations in RYR1 gene: is it more common than described? Muscle Nerve 35: 670–674. 17226826 10.1002/mus.20715

[29] Vega AV, Ramos-Mondragón R, Calderón-Rivera A, Zarain-Herzberg A, Avila G (2011) Calcitonin gene-related peptide restores disrupted excitation-contraction coupling in myotubes expressing central core disease mutations in RyR1. J Physiol 589: 4649–4669. 21825032 10.1113/jphysiol.2011.210765 PMC3213414

[30] Balshaw D, Gao L, Meissner G (1999) Luminal loop of the ryanodine receptor: a pore-forming segment? Proc Natl Acad Sci U S A 96: 3345–3347. 10097041 10.1073/pnas.96.7.3345 PMC34272

[31] Zhao M, Li P, Li X, Zhang L, Winkfein RJ, et al (1999) Molecular identification of the ryanodine receptor poreforming segment. J Biol Chem 274: 25971–25974. 10473538 10.1074/jbc.274.37.25971

[32] Gao L, Balshaw D, Xu L, Tripathy A, Xin C, et al (2000) Evidence for a role of the lumenal M3-M4 loop in skeletal muscle Ca^2+^ release channel (ryanodine receptor) activity and conductance. Biophys J 79: 828–840. 10920015 10.1016/S0006-3495(00)76339-9 PMC1300981

[33] Wang Y, Xu L, Pasek DA, Gillespie D, Meissner G (2005) Probing the role of negatively charged amino acid residues in ion permeation of skeletal muscle ryanodine receptor. Biophys J 89: 256–265. 15863483 10.1529/biophysj.104.056002 PMC1366523

[34] Ramachandran S, Serohijos AW, Xu L, Meissner G, Dokholyan NV (2009) A structural model of the pore-forming region of the skeletal muscle ryanodine receptor (RyR1). PLoS Comput Biol 5: e1000367. 19390614 10.1371/journal.pcbi.1000367 PMC2668181

[35] Xu L, Wang Y, Yamaguchi N, Pasek DA, Meissner G (2008) Single channel properties of heterotetrameric mutant RyR1 ion channels linked to core myopathies. J Biol Chem 283: 6321–6329. 18171678 10.1074/jbc.M707353200 PMC2956488

[36] Monnier N, Romero NB, Lerale J, Landrieu P, Nivoche Y, et al (2001) Familial and sporadic forms of central core disease are associated with mutations in the C-terminal domain of the skeletal muscle ryanodine receptor. Hum Mol Genet 10: 2581–2592. 11709545 10.1093/hmg/10.22.2581

[37] Du GG, Khanna VK, Guo X, MacLennan DH (2004) Central core disease mutations R4892W, I4897T and G4898E in the ryanodine receptor isoform 1 reduce the Ca^2+^ sensitivity and amplitude of Ca^2+^-dependent Ca^2+^ release. Biochem J 382: 557–564. 15175001 10.1042/BJ20040580 PMC1133812

