# Supplementary figures and images for: Ca2+ Release in Muscle Fibers Expressing R4892W and G4896V Type 1 Ryanodine Receptor Disease Mutants
Source: PLoS One. 2013 Jan 7;8(1):e54042. doi: 10.1371/journal.pone.0054042 (PMC3538700; doi:10.1371/journal.pone.0054042)

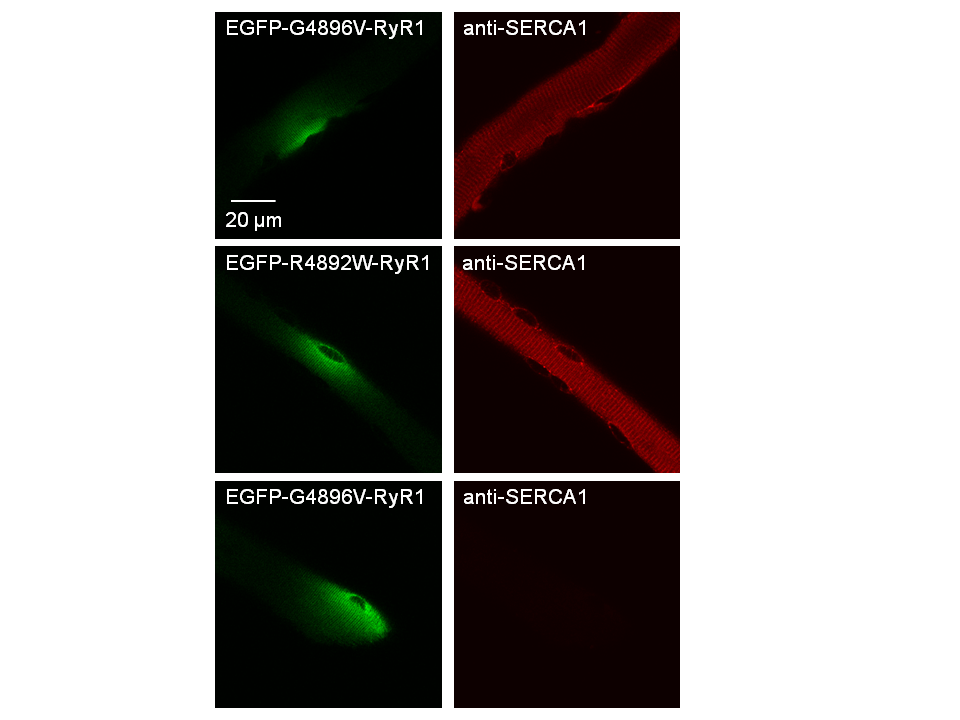

Supplement: Figure S1 — Immunofluorescence labeling of SERCA1 in fibers expressing EGFP-G4896V and EGFP-R4892W mutant RyR1 channels. Examples of local expression of either EGFP-G4896V-RyR1 (top and bottom left panels) or EGFP-R4892W-RyR1 (medium left panel) and the corresponding SERCA1 labeling. The bottom right image shows a negative control image obtained in the absence of primary antibody. (TIF) [file pone.0054042.s001.tif]
